# Therapeutical Notes

**Published:** 1899-12-23

**Authors:** 


### THERAPEUTICAL NOTES.

### Ichthyol in Phthisis.

This drug is coming into use for a great variety of purposes, since it acts either through the skin or on mucous surfaces, reducing inflammations, lessening pain and destroying bacteria, by the sulphur compounds which it forms.

In "Merck's Archives," May, 1899, a review of recent results is collected from many sources. Thus, 540 cases of phthisis were treated with ichthyol by Cohn, Scarpa, H. Fraenkel, and Le Tanneur, and the effects were most satisfactory. Goldberg found it preferable to any drug in tuberculosis of the kidney and bladder, and advises its use for a long period as he is convinced of its harmlessness.

The doses employed by these and other writers commenced with as little as two drops, but it is gradually increased up to 20, 30, or 40 drops three times a day. It may be given in a draught of water or in pills, and causes no irritation of the stomach when properly diluted, but rather aids the digestion. Combined with bismuth it gave good results in tuberculous diarrhœa, but is counter-indicated if high fever is present. It may replace for many patients either the creasote or cod liver oil which are commonly given.

For Chronic Rheumatism.

℞. Sodii iodidi, ʒiv.

Vini colchici, ʒiv.

Tinct. guiaci ammoniatæ, ʒvij.

Extr. cocæ, liq., ʒvij.

Extr. cimicifugæ liq., ʒvi.

Misce S., ʒi, ter die sumendum ex aquâ.

—*Merck's Archives*, March.

For Urticaria.

Woolff has cured many cases in 24 hours by giving sodium phosphate every three hours in doses of a drachm or more in concentrated solution. For local use we may employ—

℞. Calamini preparatæ.

Zinci oxidi, āā gr. xlv.

Acidi carbolici, gr. xv.

Aq. calcis, ℥i.

Aq. rosæ, ℥ii.

Misce ft. lotio.

A spray application is used by Gaucher :—

Alcohol.

Etheris.

Chloroformi, āā ʒiii.

Menthol, ʒi.

M. ft. lotio.

—Quoted in *Practitioner*, 1899.

Orthoform.

Kindler finds the analgesic properties of this drug valuable in gastric ulcer and carcinoma. He gives three " knife pointfuls " in a glass of water on an empty stomach, and obtains complete relief from pain in about ten minutes. This only occurs when there is an ulcerated surface, so that the orthoform can act on the exposed nerve endings. In this way it may also serve the purposes of diagnosis, as its effect in painful catarrh of the stomach is nil. In catarrhal laryngitis an insufflation only causes a saltish taste, but if ulceration is present there is first a burning feeling, and then analgesia for several hours. Though its innocuousness is remarkable, repeated applications may produce irritation, and finally some necrotic changes in the tissues. Hence it should not be given for several days together without a break.—*Medical Chronicle*, May.

Acute Colic.

℞. Tincturæ opii deodorati, ʒj.

Chloroformi, ʒjss.

Camphoræ, gr. iv.

Ol. cajuputi, ʒj.

Aquæ, ℥ij.

M. S., ʒj. omni horâ.

—*Medical Record*.

